# Comparative analysis of the complete chloroplast genomes of seven *Populus* species: Insights into alternative female parents of *Populus tomentosa*

**DOI:** 10.1371/journal.pone.0218455

**Published:** 2019-06-19

**Authors:** Dan Zong, Peihua Gan, Anpei Zhou, Jinyu Li, Zhongli Xie, Anan Duan, Chengzhong He

**Affiliations:** 1 Key Laboratory for Forest Genetic and Tree Improvement and Propagation in Universities of Yunnan Province, Southwest Forestry University, Kunming, Yunnan, China; 2 Key Laboratory of Biodiversity Conservation in Southwest China, State Forestry Administration, Southwest Forestry University, Kunming, Yunnan, China; 3 Key Laboratory for Forest Resources Conservation and Utilization in the Southwest Mountains of China, Ministry of Education, Southwest Forestry University, Kunming, Yunnan, China; National Cheng Kung University, TAIWAN

## Abstract

*Populus tomentosa*, of section *Populus*, is distributed mainly in northern China. This species has high resistance to many diseases and insects, and it plays key roles in shelterbelts and urban afforestation in northern China. It has long been suspected to be a hybrid, but its parents remain unknown. In the present study, we report four newly sequenced complete cp genomes from section *Populus* and comparative genomic analyses of these new sequences and three published cp genome sequences. The seven cp genomes ranged from 155,853 bp (*P*. *tremula* var. *davidiana*) to 156,746 bp (*P*. *adenopoda*) in length, and their gene orders, gene numbers and GC contents were similar. We analyzed SNPs, indels, SSRs and repeats among the seven cp genomes, and eight small inversions were detected in the *ndhC-trnV*, *rbcL-accD*, *petA-psbJ*, *trnW-trnP*, *rpl16-rps3*, *trnL-ycf15*, *ycf15-trnL*, and *ndhF-trnL* intergenic regions. Furthermore, seven divergent regions (*trnH-psbA*, *matK*, *psbM-psbD*, *ndhC-trnV*, *ycf1*, *ndhF-ccsA* and *ccsA-ndhD*) were found in more highly variable regions. The phylogenetic tree reveals that *P*. *tomentosa* is closely related to *P*. *alba* and *P*. *alba* var. *pyramidalis*. Hence, *P*. *alba* was involved in the formation of *P*. *tomentosa*.

## Introduction

Species of the genus *Populus*, family Salicaceae, are collectively known as poplars and cottonwoods and play important economic and ecological roles due to their rapid growth rates, easy vegetative propagation, small genome size, importance as a timber source and other features [1, 2]. The genus *Populus* is classified into 29 species belonging to six sections (*Abaso*, *Aigeiros*, *Leucoides*, *Populus*, *Tacamahaca* and *Turanga*), and it is the most widely distributed genus of woody plants in the world [3, 4]. However, because poplars readily undergo interspecific hybridization and exhibit high levels of morphological variation, the number of *Populus* species currently described in the literature varies from 22 to 85, and hundreds of *Populus* hybrids and cultivars exist [3, 5–9].

*Populus tomentosa* Carrière, known as Chinese white poplar, belongs to section *Populus*. This species is mainly distributed in northern China and has strong resistance to many diseases and insects. It also plays key roles in shelterbelts and urban afforestation in northern China. These useful features have attracted considerable attention from taxonomists and geneticists [10]. However, it is difficult to generate a large segregating population of intraspecific hybrids due to the high rate of seed abortion [11].

*P*. *tomentosa* has long been suspected to be a hybrid, but its exact parents remain unknown [12]. Based on its morphological similarity and genetic to *P*. × *hopeiensis*, *P*. *tomentosa* was thought by Wang et al [13] to have been domesticated from *P*. × *hopeiensis*, which is a sympatric with *P*. *tomentosa* in China. Additionally, because the morphological characteristics of *P*. *tomentosa* are similar to those of *P*. *canescens*, a natural hybrid of *P*. *alba* and *P*. *tremula*, Bartkowiak [14] speculated that *P*. *tomentosa* was a natural hybrid between *P*. *alba* as the female parent and *P*. *tremula* as the male parent. In addition, Zhang [15] inferred that *P*. *tomentosa* was a natural hybrid of *P*. *alba* and *P*. *tremula* var. *davidiana* based on floral characteristics.

Li et al [16] analyzed the genetic relationships among five varieties of *P*. *tomentosa* with their putative parents (*P*. *adenopoda*, *P*. *alba*, *P*. *tremula* var. *davidiana* and *P*. *tremula*) using RAPD molecular markers. They found that *P*. *tomentosa* had closer relationships with *P*. *alba* and *P*. *adenopoda* than with *P*. *tremula* var. *davidiana* and *P*. *tremula* and concluded that *P*. *tomentosa* is a natural hybrid of *P*. *alba* and *P*. *adenopoda*. However, *P*. *adenopoda* and *P*. *tomentosa* clustered in two different clades within section *Populus* in a plastid phylogeny [13]. Zhang et al [17] suggested that *P*. *tremula* var. *davidiana* and *P*. *alba* var. *pyramidalis* may have participated in the formation of some natural types of *P*. *tomentosa* based on a comparison of five related species (*P*. *adenopoda*, *P*. *alba*, *P*. *alba* var. *pyramidalis*, *P*. *tremula* var. *davidiana* and *P*. × *hopeiensis*). In addition, Kang et al [18] speculated that *P*. *alba* and *P*. *alba* var. *pyramidalis* were probably involved in the origin of *P*. *tomentosa* based on chromosomal behaviors during meiosis and pollen fertility.

Chloroplasts (cp) are inherited in a maternal manner in the majority of plants, and each of these organelles contains a quadripartite circular molecule of double-stranded DNA that comprises two inverted repeats (IRs) and two single copy regions: the large and small single copy regions (LSC and SSC) [19]. Because of their relatively small size, simple structure and conserved gene content, cpDNA sequences have been widely used for phylogenetic studies, and complete cp genome sequences could provide valuable datasets for resolving complex evolutionary relationships [20, 21]. In addition, the cp genomes have proven to be more informative than cp DNA fragments in revealing the phylogeny of land plants [20, 22–25].

In the present paper, we report four newly sequenced complete cp genomes from section *Populus* [*P*. *adenopoda* (GenBank accession number: MK341062), *P*. *alba* var. *pyramidalis* (GenBank accession number: MK341061), *P*. × *hopeiensis* (GenBank accession number: MK341060) and *P*. *tomentosa* (GenBank accession number: MK341063)] and comparative genomic analyses of the new sequences and three published cp genome sequences (*P*. *alba* (AP008956) [26], *P*. *tremula* var. *davidiana* (KX306825) [27] and *P*. *tremula* (KP861984)) [28]. The aims of our study were: (I) to reveal cp genome structure variations in *Populus* and (II) to analyze the relationships of one of the varieties of *P*. *tomentosa* with its putative female parents (*P*. *adenopoda*, *P*. *alba*, *P*. *alba* var. *pyramidalis*, *P*. *tremula* var. *davidiana*, *P*. × *hopeiensis*, and *P*. *tremula*).

## Materials and methods

### Plant material, DNA extraction, sequencing and annotation

Four accessions representing section *Populus*, comprising *P*. *adenopoda*, *P*. *alba* var. *pyramidalis*, *P*. × *hopeiensis* and *P*. *tomentosa*, were collected from Hunan (110°28'12"E, 29°07'48"N), Gansu (103°4'48"E, 38°37'12"N), Shanxi (108°4'27.95"E, 34°16'56.24"N) and Beijing (116°18'08"E, 39°57'22"N) Provinces, respectively. Chinese legislation does not forbid access to study poplar in nature reserves, so permits were not needed for samples collections, and we confirm that the samples collection did not involve endangered or protected species. Total genomic DNA was extracted from silica-dried leaves with the Ezup Plant Genomic DNA Prep Kit (Sangon Biotech, Shanghai, China).

Based on the five known *Populus* cp genome sequences (of *P*. *alba*, *P*. *balsamifera*, *P*. *euphratica*, *P*. *tremula* and *P*. *trichocarpa*) [29–31], the cp genomes of four *Populus* species were amplified with 33 primers (Table 1) using LA-PCR with Takara PrimeSTAR GXL DNA polymerase (TAKARA BIO INC., Dalian, China) following the method described by Yang [32]. A different 16 bp barcode sequences (Pacific Biosciences) was added to the primers of each of the four accessions *P*. *adenopoda*, *P*. *alba* var. *pyramidalis*, *P*. × *hopeiensis* and *P*. *tomentosa* (Table 2). The PCR products were subjected to next-generation sequencing at Nextomics Biosciences, and gaps were filled by PCR amplification and Sanger sequencing. The assembled genome sequences were preliminarily annotated in Geneious R8, and the start and stop codons were manually adjusted. The tRNA genes were further confirmed through the online tRNAscan-SE web server [33]. The gene map of the annotated *Populus* cp genome was drawn by OGdraw online [34].

**Table 1:** The 33 primers used to amplify the complete cp genome of the *Populus*.

Primer	F sequences	Primer	R sequences	Length/Kb
CP1-1-F	GGCTGAATGGTTAAAGCGCCCA	CP1-1-R	GATTAGTGCCTAGTGCGGGAAAAGC	6.0
CP1-2-F	ACCCTCTATCCTCTCTTTTTTCCAT	CP1-2-R	TTGCGTCCAATAGGATTTGAACCTATACC	6.0
CP2-1-F	GGTTCGATTCCCGCTACCCGC	CP2-1-R	TTTTGTTTCTAATTGGTCAGGGGCG	5.5
CP2-2-F	CGAAACTTAGTCAATGGTGTCCCTC	CP2-2-R	AAAGGATGGCTAGTCCAAGATGCTG	5.9
CP2-3-F	TGTACTTGAGATGCATTTCCCCTAG	CP2-3-R	GGATATGGGCTCGTGTGAGTAGGAA	6.2
CP2-4-F	TTCCTTATCGCTCAGGAAAGACAAG	CP2-4-R	TGGTTCAAATCCAGCTCGGCCC	5.5
CP3-1-F	CCCCAGTTCAAATCCGGGTGTCG	CP3-1-R	TTGTGCTTCAGGACCCCATAGTAAT	6.0
CP3-2-F	AGGGACCGTTTCGTTTTTGTGGG	CP3-2-R	ACTAACTTCGGGATTGGGCACAG	6.0
CP3-3-F	TTGTGCTATAAACGCATAAGCAGG	CP3-3-R	CGCCTTCAACCACTCGGCCA	6.5
CP4-1-F	TGTAGGAGAGATGGCCGAGTGG	CP4-1-R	AATGGGCGATGCTTGGTTACAATTT	5.0
CP4-2-F	TACGGATACCCCCAATACATCGAAA	CP4-2-R	CTTGGATACCATGAGGTGGGCCTTG	6.0
CP4-3-F	CATGGACAACTGTGTGGACCGAC	CP4-3-R	CCATTGCAATTGCCGGAAATACTAAGC	4.0
CP5-1-F	ACGGCGGGAGTCATTGGTTCA	CP5-1-R	TGAATGGCCTCGGTTAATCCTGTC	6.0
CP5-2-F	GAACTTTCGATTGATCCAGGCACT	CP5-2-R	GCATCCCCGTCGAATCAGACAGA	5.7
CP5-3-F	GCGAATCCATCAAACTCGATCAACC	CP5-3-R	AGTTCGGTAGAACATGGGTCTCCA	4.0
CP6-1-F	TGAACCTACGACATTGGGTTTTGGAGA	CP6-1-R	AAATCCAAATCGGGCAGATCTTTAC	5.5
CP6-2-F	GATCAAATTCTATCCCTTAGGAACCGT	CP6-2-R	ATTCTTTAGATCCCTTGTTTCACTCCG	6.0
CP6-3-F	GGCCGTGCAGATATATGCCTTTAAT	CP6-3-R	GCGGTGGAAAAATATGGGTACG	5.0
CP6-4-F	TTCTACCCGGTTTAACGACAGCTA	CP6-4-R	ATGTACGAGGATCCCCGCTAAGCATC	5.5
CP7-1-F	GTTTGAGCTGTACGAGATGAAATTCT	CP7-1-R	TGGGTACGTATCTTTCCCGACAA	6.0
CP7-2-F	TCTGGCTATATTTTCTGCTACCCCG	CP7-2-R	AAGATACAGGAGCGAAACAATCAACC	6.0
CP7-3-F	GCAATTCACAGGGTTCAACAAGCA	CP7-3-R	AGAGCGTGGAGGTTCGAGTCC	8.0
CP8-1-F	GGACTCGAACCTCCACGCTCT	CP8-1-R	AGAGTCCAACTCCATTGAATTGCCA	7.0
CP8-2-F	CTATATGATCCGATCGATTGCGTAAG	CP8-2-R	GCATTTCGGGGAGAACCAGCTAG	6.0
CP8-3-F	TAGGCAGTGGCTTGGTTAAGGGA	CP8-3-R	AAAAGTTGACAAACTGGGTGGATACG	6.0
CP8-4-F	CCTGACAAAATCGACGAAACGGAA	CP8-4-R	GCCGCCACTCGGACTCGAACC	4.0
CP9-1-F	GGTTCGAGTCCGAGTGGCGGC	CP9-1-R	CAGTTCTTTACTTGGGCGGTTGGA	6.0
CP9-2-F	TACCCATTGTTGTTCCAAAGACTCTAC	CP9-2-R	GGTCTAGCTTAATATCGCAAAAATGGC	5.8
CP9-3-F	TCTATGCGAGCTTTTTTCCGTATCG	CP9-3-R	ACAGCCGACCGCTCTACCAC	5.0
CP10-1-F	GTGGTAGAGCGGTCGGCTGT	CP10-1-R	CAGGGACCAGGAGATTGGATCT	6.0
CP10-2-F	GTGCTTGTTCCCCCCTTCTTCCT	CP10-2-R	CAGCATCCACCAATTCGGAACTT	6.0
CP10-3-F	CCGATATAGCAGTAAAAGCAAGACGT	CP10-3-R	TGGATCCCGCTCTAATAGCTCCG	5.0
CP10-4-F	TCTTACCTGGGATCGCAAATCCC	CP10-4-R	TGGGCGCTTTAACCATTCAGCC	5.8

**Table 2:** The barcode sequences for the five *Populus* accessions.

Species	Barcode sequences 5’	Barcode sequences 3’
*P*. *tomentosa*	GCGCGCGCACTCTCTG	CAGAGAGTGCGCGCGC
*P*. *alba var*. *pyramidalis*	TCATACACACAGATAG	CTATCTGTGTGTATGA
*P*. × *hopeiensis*	TAGTGTGCGACTCTGA	TCAGAGTCGCACACTA
*P*. *adenopoda*	TCTGTATCTCTATGTG	CACATAGAGATACAGA

### Codon usage

To examine deviations in synonymous codon usage by avoiding the influence of amino acid composition, the relative synonymous codon usage (RSCU) was detected using MEGA 5 software [35]. Because short protein-coding genes (CDS) generally result in large estimation errors for codon usage, CDS shorter than 300 bp in length were excluded from the codon usage calculations to avoid sampling bias [36]. Finally, 58 CDS for each cp genome were analyzed in this study.

### SSR and long repeat sequence analysis

Microsatellites in the seven *Populus* cp genomes were detected using MISA [37] with the minimal repeat number set to 12, 6, 5, 5, 5 and 5 for mono-, di-, tri-, tetra-, penta-, and hexa- nucleotides, respectively. All of the repeats were manually verified. We used the online REPuter software [38] to identify and locate forward repeat (F), reverse repeat (R), complemented repeat (C) and palindromic repeat (P) sequences. The following settings for repeat identification were used: (1) Hamming distance equal to 3; (2) minimal repeat size, 30 bp; and (3) maximum computed repeats, 90 bp.

### Sequence divergence analysis

To investigate divergence in cp genomes, identity across the whole cp genomes was visualized using the mVISTA viewer in the Shuffle-LAGAN mode among the seven accessions with *P*. *adenopoda* as the reference. MAFFT version 7.037 software [39] was used to align the seven cp genome sequences of *Populus*: *P*. *adenopoda*, *P*. *alba*, *P*. *alba* var. *pyramidalis*, *P*. *tremula* var. *davidiana*, *P*. × *hopeiensis*, *P*. *tomentosa* and *P*. *tremula*. After manual adjustment with BioEdit software, we performed sliding window analysis to assess variability (Pi) throughout the cp genomes using DnaSP version 5 software [40]. The window length was set to 600 bp and the step size was set to 200 bp. Single nucleotide polymorphisms (SNPs) and indels were detected using the “find variation” in Geneious R8. Inversions were manually detected using the BioEdit software. There were a total of 21 pairwise alignments for the seven cp genomes.

### Phylogenetic analysis

To detect the phylogenetic position of *P*. *tomentosa* with respect to the other *Populus* species, 14 accessions with available complete cp genomes were compared, including 10 accessions from section *Populus*, two accessions from section *Turanga* of *Populus* (*P*. *euphratica* (KJ624919) and *P*. *ilicifolia* (KX421095)) and two accessions from *Salix* (*Salix babylonica* (KT449800) and *Salix paraplesia* (MG262366)) as outgroups. The 10 cp genomes from section *Populus* included the four new cp genomes (*P*. *adenopoda*, *P*. *alba* var. *pyramidalis*, *P*. × *hopeiensis*, *P*. *tomentosa*) and six complete cp genomes published elsewhere or available from NCBI (*P*. *alba* (AP008956), *P*. *tremula* var. *davidiana* (KX306825), *P*. *qiongdaoensis* (KX534066), *P*. *rotundifolia* (KX425853), *P*. *tremula* (KP861984), and *P*. *tremula* × *alba* (MG262346)) [41–44]. The sequences were aligned using MAFFT [39] and adjusted manually where necessary. Maximum likelihood (ML) analyses were conducted using RAxML with 1000 bootstrap replicates [45]. Bayesian inference (BI) was performed using the program MrBayes 3.1.2 [46]. The jModelTest 2.0 program [47] was used to determine the best-fitting model for each dataset based on the Akaike information criterion and the optimal model of “TVM +F+R2”. The Markov chain Monte Carlo (MCMC) algorithm was run for 1,000,000 generations, and a burn-in of 25% was used for the analysis.

## Results and discussion

### Complete cp genomes of *Populus* species

**Fig 1:**
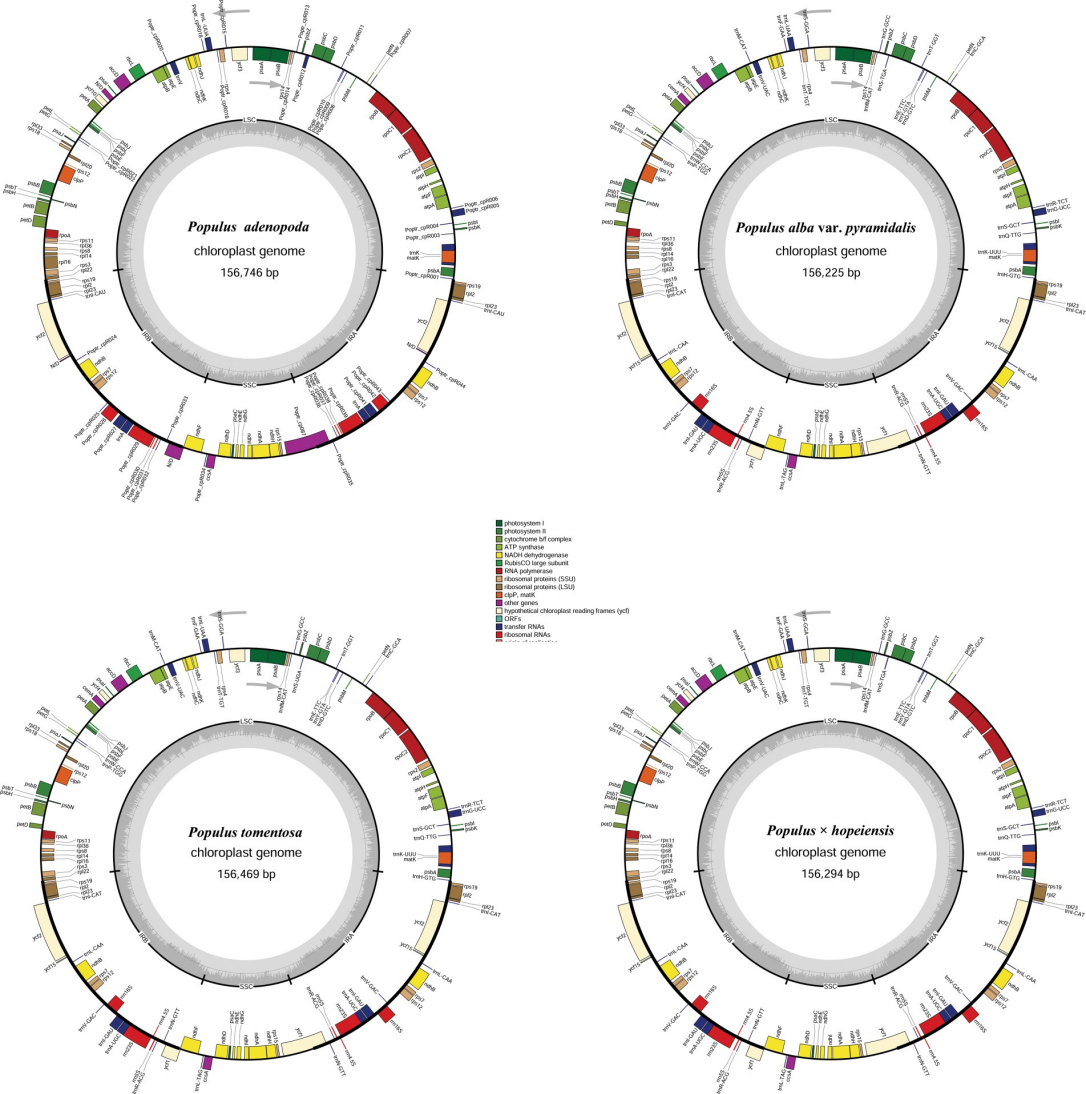
Gene map of the four *Populus* cp genomes. The genes that are drawn outside the circle are transcribed clockwise, whereas those that are drawn inside the circle are transcribed counterclockwise. The large single copy (LSC), small single copy (SSC) and inverted repeat (IRA and IRB) regions are indicated.

The seven cp genomes ranged in size from 155,853 bp (*P*. *tremula* var. *davidiana*) to 156,746 bp (*P*. *adenopoda*) (Fig 1 and Table 3). All of them displayed a typical quadripartite structure, consisting of a pair of IRs (27,571–27,660 bp) separated by the LSC (84,127–84,934 bp) and the SSC (16,413–18,584 bp) regions (Table 3). Gene content and order were very similar among the cp genomes of the seven accessions and similar to those of other published cp genomes [43, 44, 48, 49].

**Table 3:** Summary of the features of the complete cp genomes of the seven *Populus* accessions.

	*P*. *adenopoda*	*P*. *alba* [25]	*P*. *alba var*. *pyramidalis*	*P*. *tremula* var. *davidiana* [26]	*P*. × *hopeiensis*	*P*. *tomentosa*	*P*. *tremula* [27]
Size(bp)	156,746	156,505	156,225	155,853	156,294	156,469	156,067
LSC(bp)	84,934	84,618	84,431	84,127	84,629	84,722	84,377
SSC(bp)	16,552	16,567	16,530	16,584	16,413	16,531	16,490
IR(bp)	27,630	27,660	27,632	27,571	27,626	27,608	27,600
CDS(bp)	80,289	79,194	80,661	80,631	80,541	80,661	80,634
Number of total genes	130	128	130	130	130	130	130
Number of CDS genes	85(7)	83(6)	85(7)	85(7)	85(7)	85(7)	85(7)
Number of tRNA genes	37(7)	37(7)	37(7)	37(7)	37(7)	37(7)	37(7)
Number of rRNA genes	8(4)	8(4)	8(4)	8(4)	8(4)	8(4)	8(4)
Overall GC content (%)	36.79	36.74	36.79	36.76	36.73	36.78	36.76
GC content in LSC (%)	34.64	34.56	34.63	34.58	34.52	34.60	34.55
GC content in SSC (%)	30.52	30.49	30.53	30.40	30.56	30.67	30.56
GC content in IR (%)	41.97	41.95	41.97	42.01	41.95	42.00	41.97

**Fig 2:**
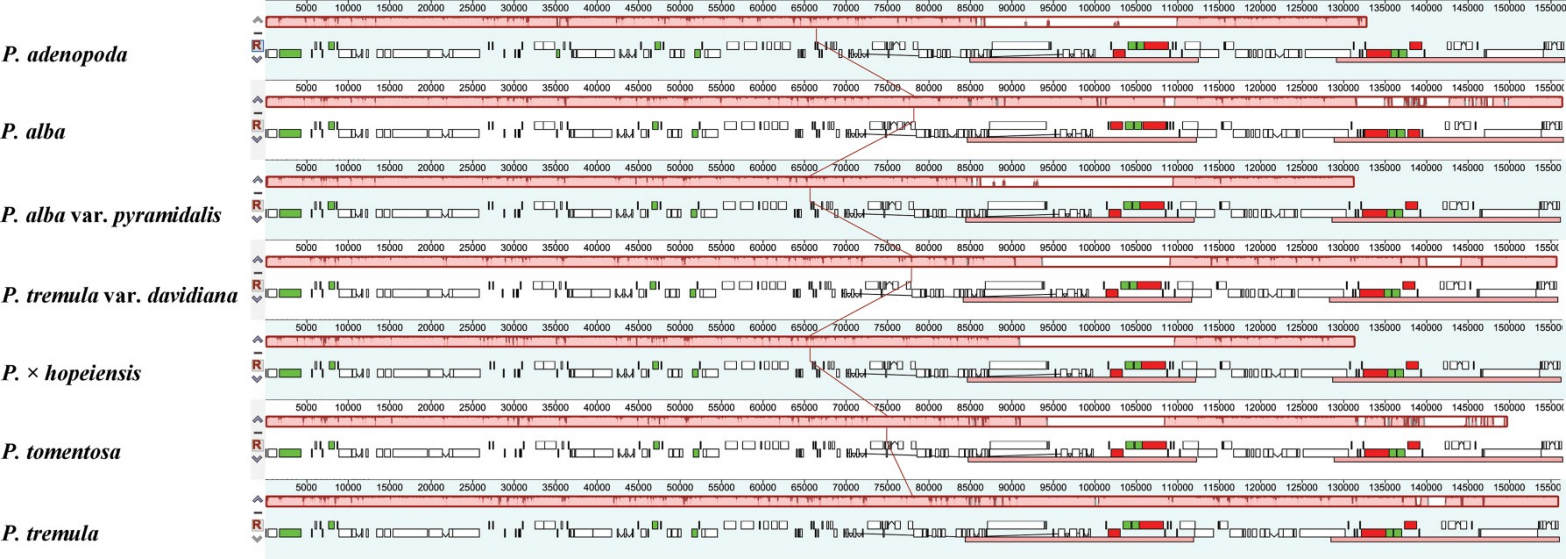
Gene arrangement map of the seven cp genomes using MAUVE software in Geneious R8. Annotations of rRNA, protein coding and tRNA genes are shown in red, white and green boxes, respectively.

The four cp genomes all encode 130 genes with the same gene order and gene clusters. Among these genes, 112 are unique genes, including 78 protein-coding genes, 30 tRNA genes and 4 rRNA genes, except in *P*. *alba*, which has 111 unique genes (77 protein-coding genes, 30 tRNA genes and 4 rRNA genes). Twelve distinct genes (*atpF*, *ndhA*, *ndhB*, *petB*, *rpl2*, *rpoC1*, *trnK-UUU*, *trnG-UCC*, *trnL-UAA*, *trnV-UAC*, *trnI-GAU*, *trnA-UGC*) contain one intron and three genes (*clpP*, *ycf3* and *rps12*) contain two introns. All annotated genes are listed in S1 Table. The overall GC contents range from 36.73% (*P*. × *hopeiensis*) to 36.79% (*P*. *adenopoda* and *P*. *alba* var. *pyramidalis*). However, the GC content is unequally distributed in the *Populus* cp genomes; it is highest in the IR regions (41.95–42.01%), moderate in the LSC regions (34.52–34.64%) and lowest in the SSC regions (30.40–30.67%) (Table 3). The IR regions have the highest GC content due to the presence of eight rRNA sequences in the IR regions [19]. Furthermore, the AT content of the seven cp genomes was 55.1%, 62.6% and 70.5% at the first, second, and third codon positions, respectively, within protein-coding positions (Table 4). Overall, these seven cp genomes show a high conservation of all genome features, such as gene content, gene order, exon-intron structure and GC content. The alignment analysis revealed that the cp genomes of the seven *Populus* accessions were highly conserved, and that no rearrangement of gene organization had occurred (Fig 2).

**Table 4:** Base composition of the CDS sequences.

		T/U	C	A	G	Length (bp)
*P*. *adenopoda*	1st position	24.0	18.5	31.1	26.5	26,763
	2nd position	33.0	19.9	29.6	17.5	26,763
	3rd position	38.0	13.9	32.4	15.8	26,763
	CDS	31.6	17.4	31.0	19.9	80,289
*P*. *alba*	1st position	24.0	18.5	31.1	26.6	26,398
	2nd position	33.0	20.0	29.6	17.6	26,398
	3rd position	38.0	13.8	32.4	15.8	26,398
	CDS	31.5	17.4	31.0	20.0	79,194
*P*. *alba var*. *pyramidalis*	1st position	24.0	18.5	31.1	26.4	26,887
	2nd position	33.0	19.9	29.6	17.6	26,887
	3rd position	38.0	13.9	32.4	15.9	26,887
	CDS	31.6	17.5	31.0	19.9	80,661
*P*. *tremula* var. davidiana	1st position	24.0	18.5	31.1	26.4	26,877
	2nd position	33.0	20.0	29.6	17.6	26,877
	3rd position	38.0	13.9	32.4	15.8	26,877
	CDS	31.6	17.5	31.0	19.9	80,631
*P*. × *hopeiensis*	1st position	24.0	18.5	31.1	26.4	26,847
	2nd position	33.0	20.0	29.6	17.6	26,847
	3rd position	38.0	13.9	32.4	15.9	26,847
	CDS	31.6	17.4	31.0	20.0	80,541
*P*. *tomentosa*	1st position	24.0	18.5	31.1	26.4	26,887
	2nd position	33.0	20.0	29.6	17.6	26,887
	3rd position	38.0	13.9	32.4	15.9	26,887
	CDS	31.6	17.5	31.0	19.9	80,661
*P*. *tremula*	1st position	24.0	18.5	31.1	26.4	26,878
	2nd position	33.0	19.9	29.6	17.6	26,878
	3rd position	38.0	13.9	32.4	15.9	26,878
	CDS	31.5	17.5	31.0	20.0	80,634

### IR expansion and contraction

**Fig 3:**
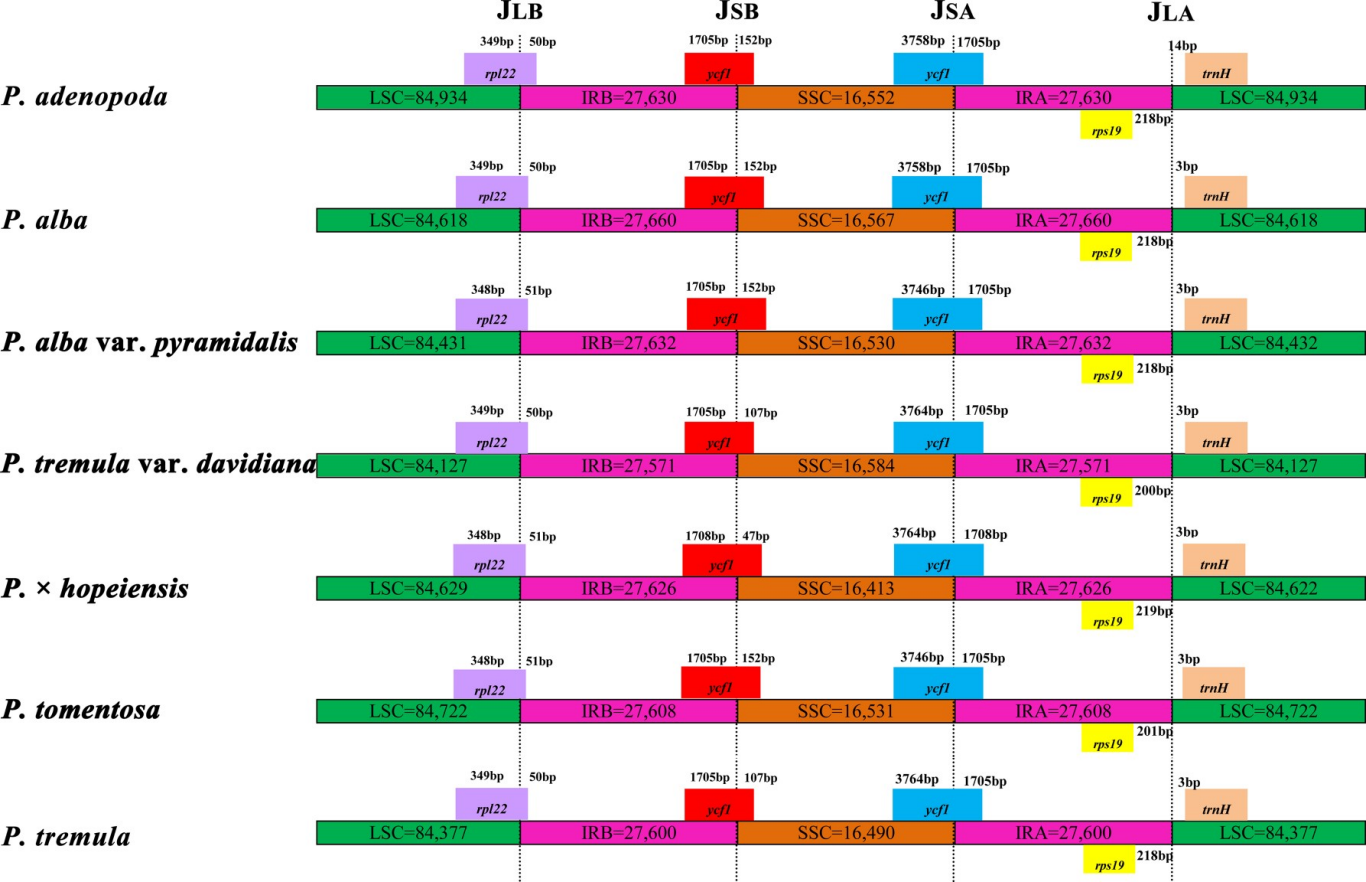
Comparison of the LSC, IR, and SSC border regions among the seven *Populus* cp genomes.

The IR regions are known to promote the stability of the other regions of the genome by intramolecular recombination between the two copies of the IRs, thus limiting recombination between the two single copy regions [50, 51]. Comparison of the boundaries between the IRs and single copy regions of the seven *Populus* accessions revealed very small boundary differences. Four junctions (J_LA_, J_LB_, J_SA_ and J_SB_) lay between the two IRs (IRB and IRA) and the two single copy regions (LSC and SSC). We carefully compared the IR border positions and the adjacent genes among the seven cp genomes. Detailed comparisons of the IR-SSC (J_SA_) and IR-LSC (J_SB_) boundaries among the cp genomes of the seven species are presented in Fig 3. For J_LA_, the boundary was located between *rps19* and the *trnH* gene. The variation in distances between *rps19* and J_LA_ was from 200 bp to 219 bp, and the distances in the four species *P*. *adenopoda*, *P*. *alba*, *P*. *alba* var. *pyramidalis* and *P*. *tremula* were the same. The distance between *trnH* and J_LA_ was consistent at 3 bp except in *P*. *adenopoda*, where it was 14 bp. The *ycf1* gene spanned the SSC and IRA regions and the *rpl22* gene spanned the LSC and IRB regions very similar length among all seven *Populus* accessions. For the *ycf1* gene, 1705 bp was integrated into the IRB region in all accessions, except *P*. × *hopeiensis*, where 1708 bp was integrated into this region, and the length of the *ycf1* gene in the SSC region was the same between the two accessions of *P*. *alba* var. *pyramidalis* and *P*. *tomentosa*.

### Codon usage

Codon usage was calculated for the protein-coding genes present in the *Populus* cp genome. Most protein-coding genes employ the standard ATG as the initiator codon. However, ATA, ATC, TTC, and ATT are also used as alternative start codons [52]. Among the *Populus* protein-coding genes, two genes were used as alternative start codons ATC for *rpl16* and GTG for *ndhD*.

The codon usage patterns of the 58 distinct protein-coding genes in the seven cp genomes were examined. The cp genomes of *P*. *alba* var. *pyramidalis* and *P*. *tomentosa* were consistent, each with a length of 75,960 bp and containing 25,320 codons, whereas those of *P*. *adenopoda*, *P*. *alba*, *P*. *tremula* var. *davidiana*, *P*. × *hopeiensis*, *P*. *tremula* were 75,588 bp, 74,778 bp, 75,930 bp, 75,840 bp and 75,933 bp, respectively, in size and contained 25,196, 24,926, 25,310, 25,280 and 25,311 codons, respectively, as shown in S2 Table.

**Fig 4:**
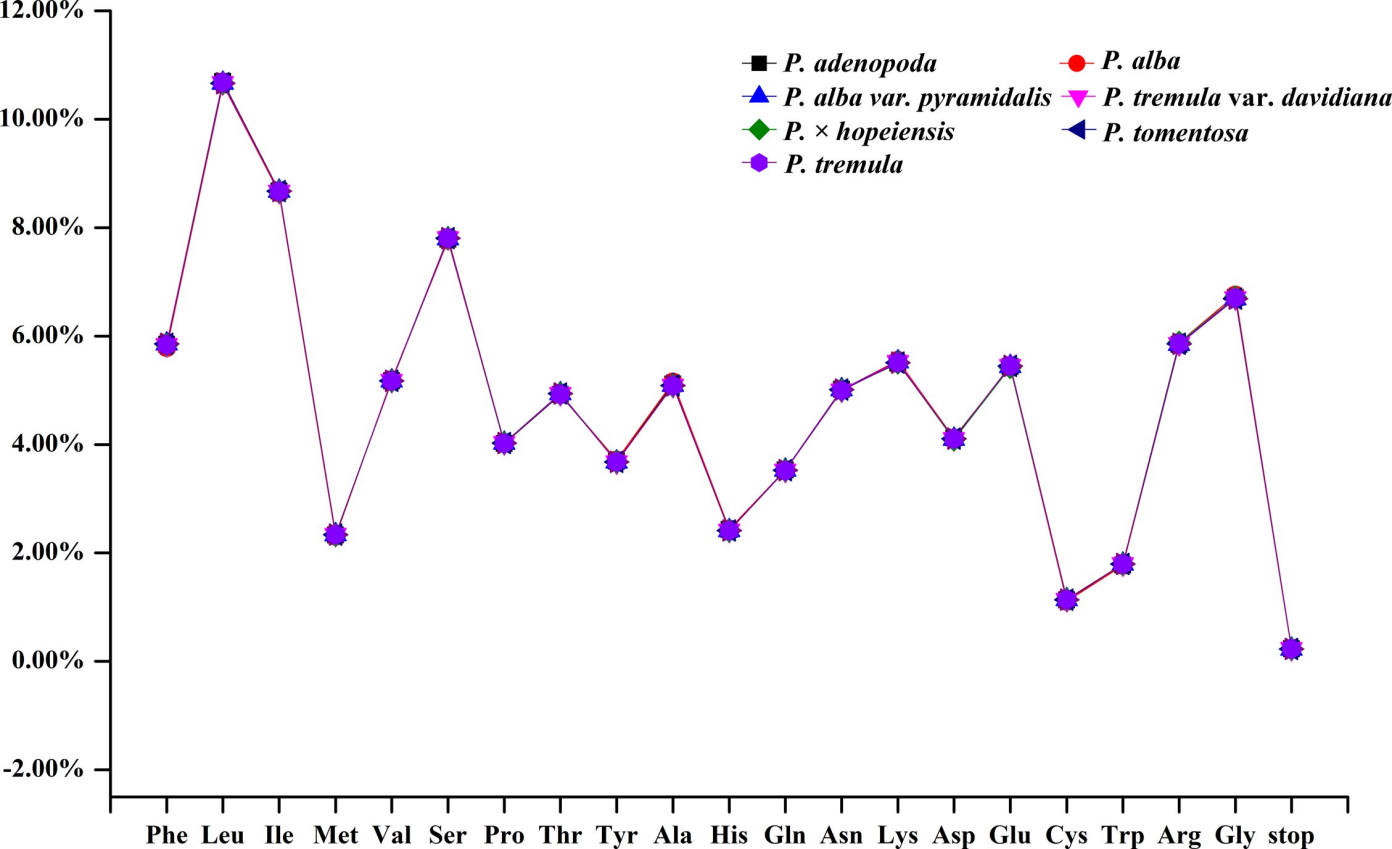
Amino acid frequencies of the seven *Populus* cp genomes based on 58 protein-coding sequences.

As an important indicator of codon usage bias, the RSCU value is the frequency observed for a codon divided by its expected frequency [53]. The values are divided into four categories: RSCU value of less than 1.0 (lack of bias), RSCU value between 1.0 and 1.2 (low bias), RSCU value between 1.2 and 1.3 (moderate bias) and RSCU value greater than 1.3 (high bias) [54, 55]. Our results showed that the RSCU values corresponding to the usage of 31 codons in the seven accessions showed preferences (<1) for except methionine (Met) and tryptophan (Trp), with 29 codons having A/T in the third codon position. All three stop codons were present, with UAA being the most frequent stop codon in all seven cp genomes (S2 Table). In addition, our results indicated that leucine (Leu: 10.70%, 10.65%, 10.67%, 10.67%, 10.67%, 10.66%, 10.66% and 10.68%) and cysteine (Cys: 1.13%, 1.11%, 1.14%, 1.14%, 1.14%, 1.14%, 1.14% and 1.14%) were the most and least commonly encoded amino acids, respectively, in all seven cp genomes (Fig 4 and S2 Table).

### SSR and long repeat analyses

**Fig 5:**
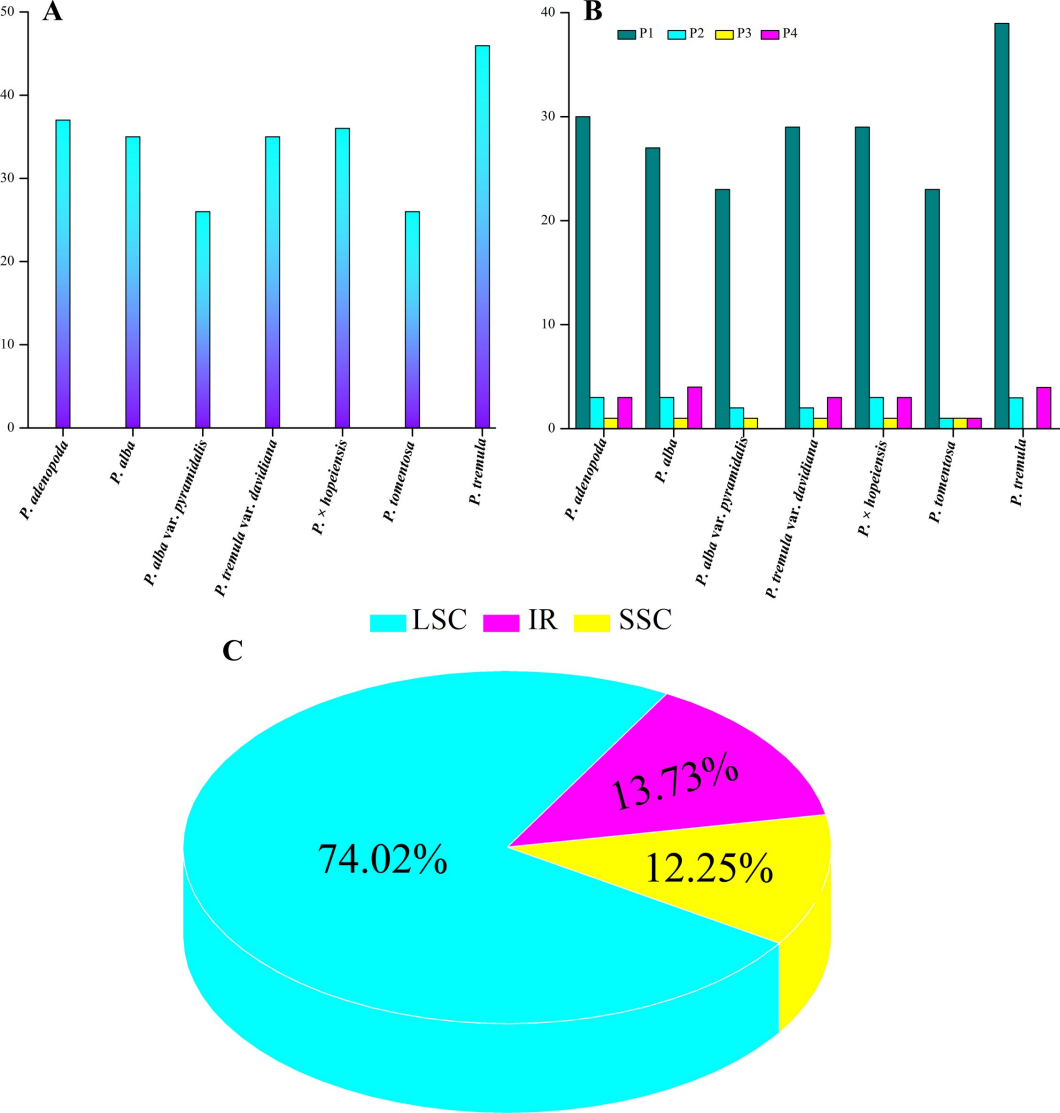
Comparison of SSRs among the seven cp genomes. **(**A) The number of SSRs detected in seven *Populus* cp genomes. (B) The number of SSR types detected in seven *Populus* cp genomes: P1, mononucleotide repeat; P2, dinucleotide repeat, P3, trinucleotide repeat and C, compound repeat (C) Frequencies of identified SSRs.

Simple sequence repeats (SSRs) can be used as genetic markers in population genetics and evolutionary studies of closely related species, because of their high variability at the intraspecific level [56–58]. The number of cp genomes SSRs (cpSSRs) ranged from 26 to 46 among the seven *Populus* accessions (Fig 5A). The number of cpSSRs in *P*. *tomentos*a (26) was the same as that in *P*. *alba* var. *pyramidalis* (26), and the numbers of cpSSRs in the three accessions *P*. *alba*, *P*. *tremula* var. *davidiana* and *P*. × *hopeiensis* were similar (S3 Table). The mononucleotide repeat (P1) number with the highest variability ranged from 23 (*P*. *alba* var. *pyramidalis* and *P*. *tomentosa*) to 39 (*P*. *tremula*), and all of the P1s were composed of poly A (polyadenine) and poly T (polythymine) repeats (Fig 5B and S3 Table). Research has shown that, in the cp genome, SSRs are generally composed of polythymine (poly T) or polyadenine (poly A) repeats and infrequently contain tandem cytosine (C) and guanine (G) repeats [59, 60]. In addition, all the dinucleotide repeat (P2) sequences in the seven accessions were AT repeats. In total, 74.83% SSRs were detected in the LSC region, 13.85% in the IR regions and 12.12% in the SSC region (Fig 5C). In general, the cpSSRs of the seven *Populus* accessions represented abundant variation and will be useful for assays detecting polymorphisms at the population level for inferring distant phylogenetic relationships among *Populus* species [61].

**Fig 6:**
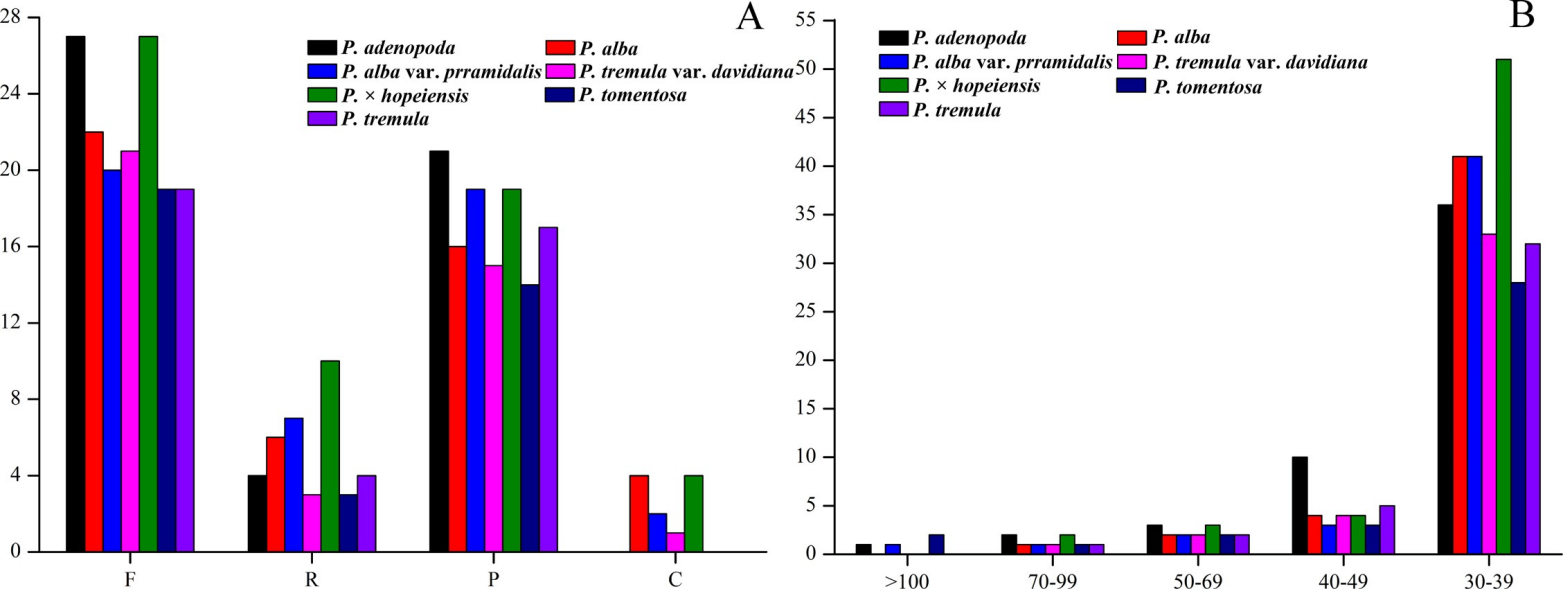
Analysis of repeat sequences in the seven cp genomes. (A) Frequency of repeat types. Forward repeat (F), reverse repeat (R), palindrome repeat (P) and complement repeat (C). (B) Frequency of repeats ≥30 bp in length.

Long repeat sequences have important roles in cp genome evolution and genome rearrangements and can be informative in phylogenetic studies [62]. Four repeat types were detected in the cp genome using REPuter software. However, complement repeats (C) were only identified in *P*. *alba*, *P*. *alba* var. *pyramidalis*, *P*. *tremula* var. *davidiana* and *P*. × *hopeiensis* which had four, two, one and four repeats, respectively. Nineteen forward repeats (F), three reverse repeats (R) and 14 palindrome repeats (P) were discovered in *P*. *tomentosa*. The repeat numbers of the other six cp genomes are shown in Fig 6A and S4 Table. The repeats were mostly distributed in the intergenic spacer (IGS) and intron sequences (S4 Table). Among these repeats, 290 (80.78%) had lengths of 30–39 bp, and only five (1.39%) were longer than 100 bp (Fig 6B). The presence of these repeats indicates that the locus is a crucial hotspot for genome reconfiguration [63, 64]. Furthermore, these repeats are an informative source for developing genetic markers for phylogenetic and population studies [64].

### Genome variation

SNP markers are the most abundant type of mutation and the most important marker for species identification [65]. Indels not only play an important role in elucidating genome evolution [66, 67], but also have potential value in constructing phylogenies [68, 69]. In this study, we compared these polymorphisms among the seven cp genomes. The numbers of nucleotide substitutions and indels varied from 44 to 274 and 129 to 252, respectively, and most mutations were located in noncoding regions (Table 5, S5 and S6 Tables).

**Table 5:** Numbers of nucleotide substitutions and indels in the seven cp genomes.

	*P*. *adenopoda*	*P*. *alba*	*P*. *alba* var. *pyramidalis*	*P*. *tremula* var. *davidiana*	*P*. × *hopeiensis*	*P*. *tomentosa*	*P*. *tremula*
*P*. *adenopoda*	/	143	155	225	249	173	243
*P*. *alba*	163(85/78)	/	135	220	245	149	244
*P*. *alba* var. *pyramidalis*	150(76/74)	74(33/41)	/	216	234	129	239
*P*. *tremula* var. *davidiana*	260(139/121)	259(139/120)	250(131/119)	/	155	234	223
*P*. × *hopeiensis*	262(134/128)	259(139/120)	246(128/118)	124(64/60)	/	246	250
*P*. *tomentosa*	151(83/68)	77(36/41)	44(20/24)	258(140/118)	258(138/120)	/	252
*P*. *tremula*	259(138/121)	255(139/116)	214(132/82)	274(148/126)	259(136/126)	254(137/117)	/

The lower triangle shows the numbers of nucleotide substitutions in complete cp genomes, and the numbers of Ts and Tv are presented in parentheses. The upper triangle indicates the numbers of indels.

In searching for SNPs and indels, we found little differences among the cp genome sequences of *P*. *tomentosa*, *P*. *alba* and *P*. *alba* var. *pyramidalis*, which had similar mutation models (S5 and S6 Tables). Interestingly, there were always more transitions (Ts) than transversions (Tv) in *Populus* except in two pairwise comparisons (*P*. *tomentosa* vs. *P*. *alba* and *P*. *tomentosa* vs. *P*. *alba* var. *pyramidalis*). Transitions (Ts) occurred at higher frequencies than did transversions (Tv) in almost all DNA sequences; transition/transversion (Ts/Tv) bias is a general property of DNA sequence evolution [70]. In the gene coding regions, seven genes (*atpB*, *ndhD*, *ndhF*, *rpoB*, *rpoC2*, *rps8* and *ycf1*) were found to have SNP mutations, and four of genes had more synonymous substitutions than nonsynonymous substitutions between *P*. *alba* var. *pyramidalis* and *P*. *tomentosa* (S7 Table). In addition, 13 genes had SNP mutations between *P*. *tomentosa* and *P*. *alba* (S7 Table). Therefore, the phylogenetic relationships of these species may be affected by different mutation models.

### Small inversions

Small inversions in the cp genomes of angiosperms are ubiquitous and are commonly associated with a hairpin secondary structure [71, 72]. Small inversions are generally detected by performing pairwise comparisons between sequences of closely related taxa [71]. In this study, a total of eight small inversions were uncovered based on the sequence alignment of the seven complete cp genomes, of which five were located in the LSC region, two were located in the IR regions, and one was located in the SSC region. In addition, eight small inversions were detected in the *ndhC-trnV*, *rbcL-accD*, *petA-psbJ*, *trnW-trnP*, *rpl16-rps3*, *trnL-ycf15*, *ycf15-trnL* and *ndhF-trnL* intergenic regions. The number of small inversions among the 21 pairwise alignments ranged from one to six. There was one small inversion between *P*. *alba* var. *pyramidalis* and *P*. *tomentosa* located in *petA-psbJ* and one between *P*. *tremula* var. *davidiana* and *P*. *tremula*, which was located in *ndhF-trnL* (S8 Table).

#### Genome sequence divergence among the seven *Populus* accessions

**Fig 7:**
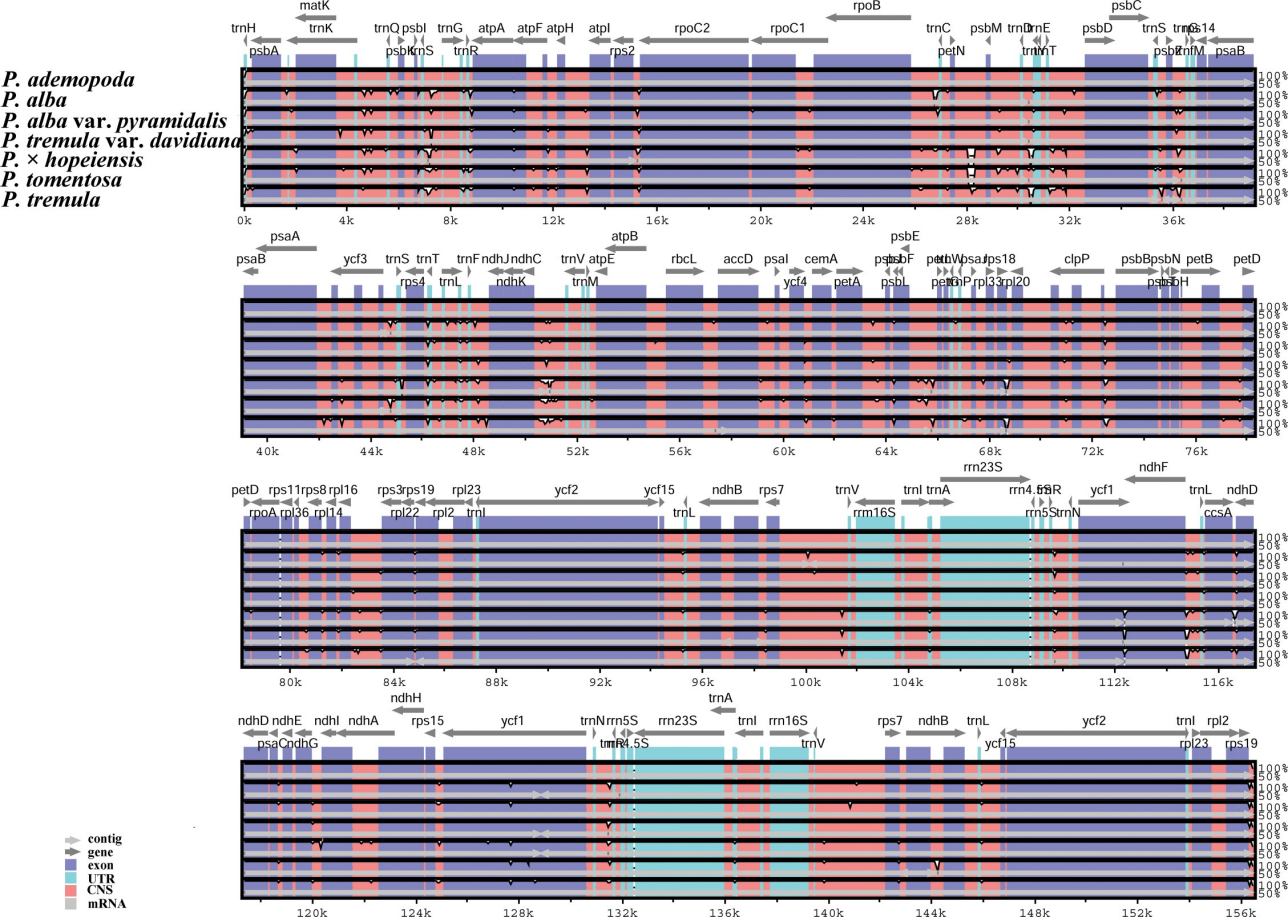
Whole cp genome alignments of the seven *Populus* accessions using the mVISTA program, with *P*. *adenopoda* as the reference. The Y-axis indicates identity from 50% to 100% and gray arrows indicate the position and direction of each gene. Red indicates noncoding sequences (CNS), blue indicates the exons of protein-coding genes (exon) and green indicates tRNA or rRNA genes.

**Fig 8:**
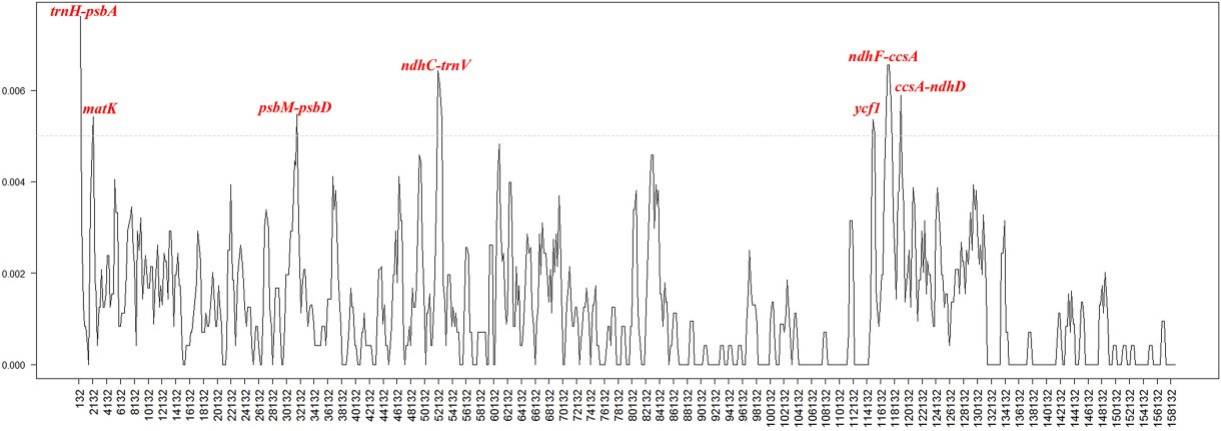
Comparison of the nucleotide variability values of the seven cp genomes of *Populus*. The genetic divergences among the *Populus* cp genomes were calculated with the DnaSP software (window length, 600 bp; step size, 200 bp). X-axis, position of the midpoint of a window; Y-axis, nucleotide diversity of each window.

We used mVISTA to perform a sequence identity analysis with *P*. *adenopoda* as a reference (Fig 7). The alignment revealed high sequence similarity across the cp genomes, which suggests that they are highly conserved. To investigate the levels of sequence divergence, we calculated the levels of genetic divergence among the cp genomes of the seven accessions using DnaSP software. The pairwise nucleotide divergence values between two of the seven cp genomes varied from 0.00028 to 0.00164 (Table 6), with a mean of 0.00103. Using sliding window analysis, we identified the seven most divergent regions (Pi>0.005): *trnH-psbA*, *matK*, *psbM-psbD*, *ndhC-trnV*, *ycf1*, *ndhF-ccsA*, and *ccsA-ndhD* (Fig 8). Further work is necessary to determine whether these seven variable loci can be used in *Populus* phylogenetic analyses or serve as excellent candidate markers for population genetic and phylogenetic analysis [73].

**Table 6:** Pairwise nucleotide divergences of the eight cp genomes of *Populus*.

	*P*. *adenopoda*	*P*. *alba*	*P*. *alba* var. *pyramidalis*	*P*. *tremula* var. *davidiana*	*P*. *hopeiensis*	*P*. × *tomentosa*	*P*. *tremula*
*P*. *adenopoda*	/						
*P*. *alba*	0.00066	/					
*P*. *alba* var. *pyramidalis*	0.00063	0.00031	/				
*P*. *tremula* var. *davidiana*	0.00111	0.00110	0.00106	/			
*P*. × *hopeiensis*	0.00114	0.00113	0.00109	0.00054	/		
*P*. *tomentosa*	0.00064	0.00032	0.00028	0.00108	0.00112	/	
*P*. *tremula*	0.00112	0.00110	0.00108	0.00118	0.00121	0.00164	/

### Phylogenetic analysis

Cp genomes provide abundant resources, that are useful for evolutionary, taxonomic, and phylogenetic studies [20, 60, 74]. Whole cp genomes and protein-coding genes have been successfully used to resolve phylogenetic relationships at almost every taxonomic level during the past decade [60, 75].

**Fig 9:**
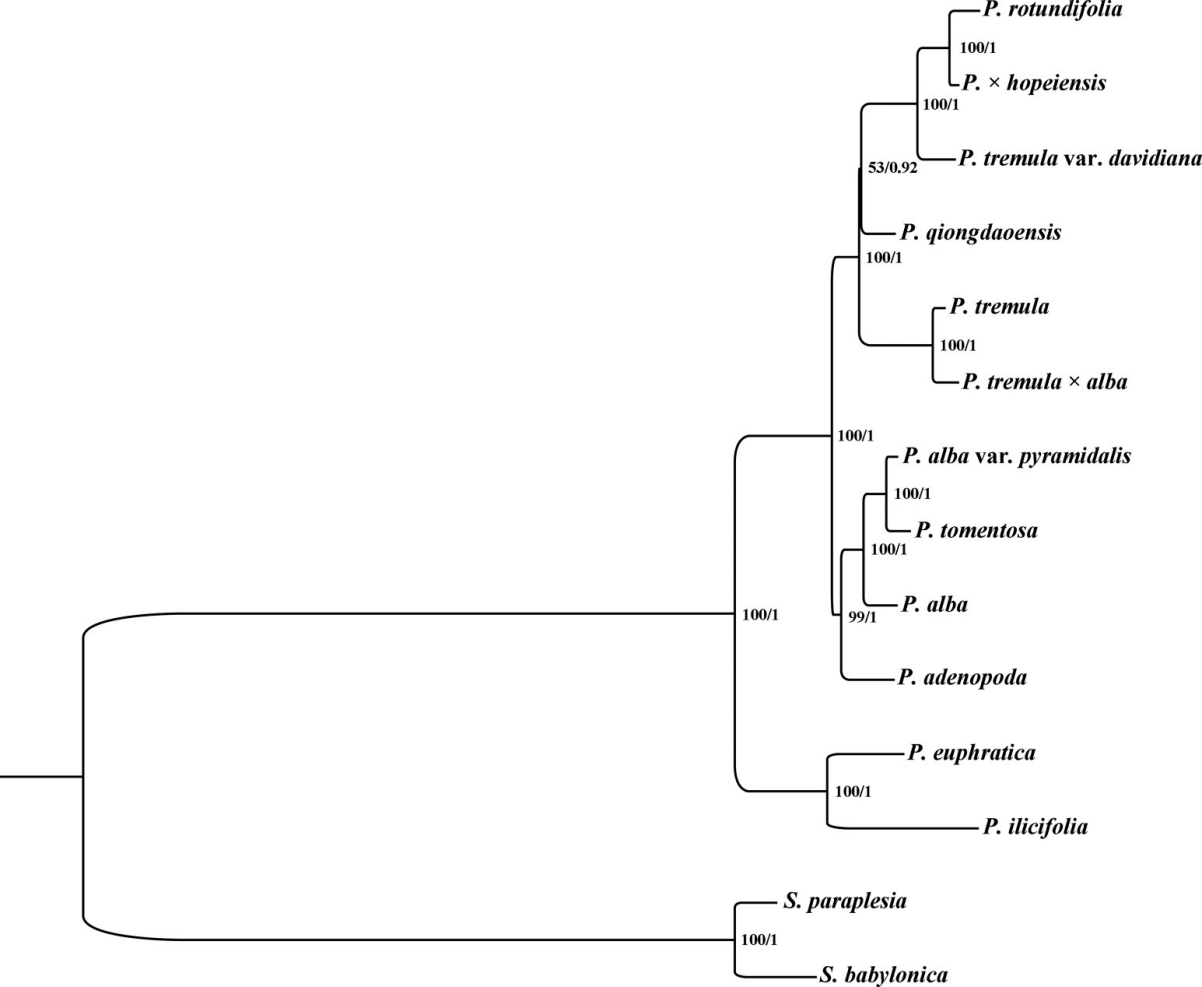
Phylogeny of the 12 *Populus* species inferred from ML and BI analysis of the cp genome dataset. Numbers between the lines on the left indicate the ML bootstrap values for clades with >50% support, and the numbers on the right indicate the Bayesian posterior probabilities.

The complete cp genome sequences of the seven *Populus* accessions and the three published complete cp genomes of members of section *Populus* (*P*. *qiongdaoensis* (KX534066), *P*. *rotundifolia* (KX425853), *P*. *tremula* × *alba*) were used for phylogenetic analysis, with *P*. *euphratica* (KJ624919), *P*. *ilicifolia* (KX421095), *Salix babylonica* (KT449800) and *Salix paraplesia* (MG262366) included as outgroups. ML and BI nucleic acid analyses were performed, and the results are summarized in Fig 9. The two topologies show similar phylogenetic patterns. Each topology divided the 10 *Populus* accessions into two clades. The first divergent clade contained *P*. *adenopoda*, *P*. *alba*, *P*. *alba* var. *pyramidalis*, and *P*. *tomentosa*, and the second contained *P*. *rotundifoli*a, *P*. × *hopeiensis*, *P*. *tremula* var. *davidiana*, *P*. *qiongdaoensis*, *P*. *tremula*, and *P*. *tremula* × *alba*. The phylogenetic tree revealed that *P*. *tomentosa* was closely related to both *P*. *alba* var. *pyramidalis* and *P*. *alba* (bootstrap support = 100% and BI = 1.0).

According to the published Flora of China [76], the species of section *Populus* share a smooth bark but vary in bark color. In *P*. *tomentosa*, the color of the bark on the basal part of the trunk changes with plant age, shifting from dull gray to grayish green or grayish white and then to dark gray. The bark is grayish white in *P*. *alba* and *P*. *adenopoda*, grayish green or grayish white in *P*. *tremula* var. *davidiana* and *P*. *tremula*, and yellowish green to grayish white in *P*. × *hopeiensis*. In addition, the shape of the bud in *Populus* is ovoid or ovoid-globose and the shape of capsule is narrowly conical or long ovoid-ellipsoid; only *P*. *tomentosa*, *P*. *alba* and *P*. × *hopeiensis* have dense, white tomentose buds. In a previous analysis of bract and other characteristics, of the 22 *P*. *tomentosa* clones, the clones were divided into three populations, and the authors speculated that *P*. *tomentosa* is a natural hybrid of *P*. *alba* and *P*. *tremula* var. *davidiana* [15]. Zhang et al [17] compared 26 traits among *P*. *tomentosa* and five related species (including several varieties). Their comparisons revealed that *P*. *tremula* var. *davidiana* and *P*. *alba* var. *pyramidalis* may have participated in the formation of some natural types of *P*. *tomentosa*, although substantial variation exists among natural types. In addition, although many wild *P*. *tomentosa* ecotypes have arisen during the evolution of the species [77], 14 clones collected from throughout the species’ entire natural ranges, clustered together in an amplified fragment length polymorphism (AFLP) marker analysis [78].

Wang et al [79] used 24 single-copy nuclear DNA sequences and 12 plastid fragments to reconstruct the phylogeny of *Populus*, which suggested that section *Populus* is a monophyletic group. The genus was divided into two distinct clades with maximum bootstrap support and posterior probability. The nuclear DNA phylogeny revealed a close relationship between *P*. *tomentosa* and *P*. *adenopoda*; however, in the plastid phylogeny, *P*. *tomentosa* and *P*. *adenopoda* belonged to two different clades. The authors speculated that in the hybridization event giving rise to *P*. *tomentosa*, the ancestor of *P*. *tremula* var. *davidiana* and *P*. × *hopeiensis* served as the maternal parent and *P*. *adenopoda* served as the paternal role. To clarify the origins of *P*. *tomentosa* and *P*. × *hopeiensis*, Wang et al [80] analyzed 10 nuclear DNA sequences and 6 cpDNA sequences from 392 individuals from 36 populations of 8 taxa (*P*. × *hopeiensis*, *P*. *tomentosa*, *P*. *alba*, *P*. *adenopoda*, *P*. *tremula* var. *davidiana*, *P*. *tremula*, *P*. *tremuloides*, and *P*. *grandidentata*). The authors aimed to improve the understanding of hybridization and introgression in section *Populus*. The results supported the division of *P*. *tomentosa* into two genetic types (mb1 and mb2) with different maternal parents; in both genetic types, *P*. *alba* acted as the male parent, whereas *P*. *adenopoda* and *P*. *tremula* var. *davidiana* acted as the maternal parent in mb1 and mb2, respectively. However, there is always a big controversy about the possible parent of *P*. *tomentosa*. RAPD and AFLP analyses have suggested that *P*. *tomentosa* is possibly a natural hybrid of *P*. *alba* and *P*. *adenopoda* [14–15], an interpretation highly consistent with observations of chromosomal behaviors during meiosis and pollen fertility [18]. Our analyses showed that *P*. *alba* var. *pyramidalis* is closely related *P*. *tomentosa*, and Yin [81] suggested that *P*. *alba* var. *pyramidalis* could be regarded as a variant of *P*. *alba*. Based on these findings, we speculate that *P*. *alba* was involved in the formation of *P*. *tomentosa* as a common female parent based on the cp genomes.

## Conclusion

In the present study, we report four newly sequenced complete cp genomes from section *Populus* and comparative genomic analyses of these genomes and three other published cp genome sequences. The seven cp genomes were similar in structure and had a high degree of synteny. Comparison of seven cp genomes revealed seven divergent regions (*trnH-psbA*, *matK*, *psbM-psbD*, *ndhC-trnV*, *ycf1*, *ndhF-ccsA* and *ccsA-ndhD*) in the highly variable regions, which can be utilized as potential molecular markers for population genetic and phylogenetic studies in *Populus*. The location and distribution of SSRs and long repeat sequences were examined and shown to be similar and conserved among the genomes. In addition, a total of seven small inversions were detected in the *ndhC-trnV*, *rbcL-accD*, *petA-psbJ*, *trnW-trnP*, *rpl16-rps3*, *trnL-ycf15*, *ycf15-trnL* and *ndhF-trnL* intergenic regions. ML and BI phylogenetic trees based on the complete cp genome sequences indicated that *P*. *tomentosa* is closely related to both *P*. *alba* var. *pyramidalis* and *P*. *alba*. Thus, we speculate that *P*. *alba* was involved in the formation of *P*. *tomentosa* as a common female parent.

## Supporting information

S1 Table List of genes in the cp genomes. (XLSX)

S2 Table Codon usage in the eight *Populus* cp genomes. (XLSX)

S3 Table SSR repeats in the eight cp genomes. (XLSX)

S4 Table Repeat sequences in the eight cp genomes. (XLSX)

S5 Table Transitions (Ts) and transversions (Tv) among the 28 pairwise alignments. (XLSX)

S6 Table Indels among the 28 pairwise alignments. (XLSX)

S7 Table Comparisons of synonymous (S) and nonsynonymous (N) substitutions per gene of protein-coding chloroplast genes among the 21 pairwise alignments. (XLSX)

S8 Table Small inversions among the 21 pairwise alignments. (XLSX)

## References

[1] Stettler RF, Zsuffa L, Wu R. The role of hybridization in the genetic manipulation of *Populus* In Stettler RF, Bradshaw HD, Heilman PE, Hinckley TM [eds]. Biology of *Populus* and its implications for management and conservation. NRC Research Press, National Research 1996

[2] Cervera MT, Storme V, Soto A, Ivens B, Van Montagu M, Rajora OP, et al Intraspecific and interspecific genetic and phylogenetic relationships in the genus *Populus* based on AFLP markers. Theor Appl Genet 2005; 111: 1440–1456. 10.1007/s00122-005-0076-2 16211377

[3] Eckenwalder JE. Systematics and evolution of *Populus*, in: Stettler RF, Bradshaw HD, Heilman PE. Hinckley TM. (Eds). Biology of *Populus* and its implications for management and conservation, National Research Council of Canada, Ottawa, Ontario, Canada, NRC Research Press 1996; 7–32.

[4] Hanzeh M, Dayanandan S. Phylogeny of *Populus* (Salicaceae) based on nucleotide sequences of chloroplast *trnT-trnF* region and nuclear rDNA. Am. J. Bot. 2004; 91: 1398–1408. 10.3732/ajb.91.9.1398 21652373

[5] Eckenwalder JE. North American cottonwoods (*Populus*, Salicaceae) of sections *Abaso*, and *Aigeiros*. J. Arnold Arboretum. 1977a; 58: 193–208.

[6] Eckenwalder JE. Systematics of Populus L. in southwestern North American with special reference to sect. Aigeiros Duby, Ph.D thesis University of California, Berkeley, CA. 1977b.

[7] Dickmann DI, Stuart K. The culture of poplars in Eastern North America. Rhodora. 1983; 19: 10–15.

[8] Wang YZ. Gilbert MG. Stellera Linnaeus In: Wu CY, Raven PH. (Eds.). Flora of China. Science Press, Beijing, 2007.

[9] Wan XQ, Zhang F, Zhong Y, Ding YH, Wang LW, Hu TX. Study of genetic relationships and phylogeny of the native *Populus* in southwest China based on nucleotide sequences of chloroplast *trnT-trnF* and nuclear DNA. Plant Syst. Evol. 2013; 299: 57–65.

[10] Zhu ZT. Collection, conservation and utilization of plus tree resources of *Populus tomentosa* in China. J. Beijing Forestry University. 1992; 14(3): 1–25.

[11] Zhang D, Zhang Z, Yang K, Li B. Genetic mapping in (*Populus tomentosa* × *Populus bolleana*) and *P*. *tomentosa* Carr. using AFLP markers. Theor. Appl. Genet. 2004; 108: 657–662. 10.1007/s00122-003-1478-7 14564399

[12] Wang DS. Molecular phylogeny of section *Leuce* and the hybeidization origin of hybrids in section *Leuce* of *Populus*. 2016; Chinese Academy of Forestry.

[13] Wang ZS, Du SH, Dayanandan S, Wang DS, Zeng YF. Phylogeny reconstruction and hybrid analysis of *Populus* (Salicaceae) based on nucleotide sequences of multiple single-copy nuclear genes and plastid fragments. PLoS ONE. 2014; 9: e103645 10.1371/journal.pone.0103645 25116432 PMC4130529

[14] Bartkowiak S. Floral bractlets in poplars of the section leech duby as a diagnostic feature. Arboretum Kornickie 1958; 3: 221–236.

[15] Zhang T.Z. Studies on the floral variafion of *Populus tomentosa* clones and their populations. J. Northwest Forestry College. 1995; 10(1): 43–47.

[16] Li KY, Huang MR, Wang MX. Study on origin of *Populus tomentosa* carr. Acta Phytotaxonomica Sinica. 1997; 35: 24–31.

[17] Zhang J, Jiang JM. A numerical taxonomic study on morphological characters of *Populus tomentosa* on its relative species. Forest Research 1991; 4: 86–90.

[18] Kang XY, Zhu ZT, Zhang ZY. Cytogenetic studies on the origin of Chinese white poplar. Journal of Beijing Forestry University. 1999; 21: 6–10.

[19] Huotari T, Korpelainen H. Complete chloroplast genome sequence of *Elodea Canadensis* and comparative analysis with other monocot plastid genomes. Gene 2012; 508: 96–105. 10.1016/j.gene.2012.07.020 22841789

[20] Jansen RK, Cai ZQ, Raubeson LA, Daniell H, dePamphilis CW, Leebens-Mack J, et al Aanalysis of 81 genes from 64 plastid genomes resolves relationships in angiosperms and identifies genome scale evolutionary patterns. Proc. Natl. Acad. Sci. USA. 2007; 104: 19369–19374. 10.1073/pnas.0709121104 18048330 PMC2148296

[21] Moore MJ, Soltis PS, Bell CD, Burleigh JG, Soltis DE. Phylogenetic analysis of 83 plastid genes further resolves the early diversification of eudicots. Proc. Natl. Acad. Sci. USA. 2010; 107: 4623–4628. 10.1073/pnas.0907801107 20176954 PMC2842043

[22] He LX, Suo ZL, Zhang CH, Jin XB, Zhao DX, Zhao XQ, et al Classification of Chinese medicinal tree peony cultivars based on chloroplast DNA sequences. AASRI Procedia 1, 2012; 344–352.

[23] Suo ZL, Zhang CH, Zheng YQ, He LX, Jin XB, Hou BX, et al Revealing genetic diversity of tree peonies at micro-evolution level with hypervariable chloroplast markers and floral traits. Plant Cell Rep. 2012; 31, 2199–2213. 10.1007/s00299-012-1330-0 22961193

[24] Dong WP, Xu C, Li DL, Jin XB, Lu Q, Suo ZL. Comparative analysis of the complete chloroplast genome sequences in psammophytic *Haloxylon* species (Amaranthaceae). PeerJ. 2016; 4, e2699 10.7717/peerj.2699 27867769 PMC5111891

[25] Xu C, Dong WP, Li WQ, Lu YZ, Xie XM, Jia XB, et al Comparative analyses of six *Lagerstroemia* complete chloroplast genomes. Front. Plant Sci. 2017; 8: 15 10.3389/fpls.2017.00015 28154574 PMC5243828

[26] Okumura S, Sawada M, Shimamura M, Park YW, Hayashi T, Yamashita A, et al A strategy for desert afforestation using plastid transformation technique for CO_2 sequestration. Journal of arid land studies. 2006; 15: 506–508.

[27] Choi MN, Han M, Park HS, Kim MY, Kim JS, Na YJ. The complete chloroplast genome sequence of *Populus davidiana* Dobe. Mitochondrial DNA B Resour. 2016; 1: 674–675. 10.1080/23802359.2016.1219634 PMC7800723 33473593

[28] Kersten B, Faivre Rampant P, Mader M, Le Paslier MC, Berard A, Vettori C, et al Genome sequences of *Populus tremula* chloroplast and mitochondrion: Implications for holistic poplar breeding. PLoS ONE; 2016; e0147209 10.1371/journal.pone.0147209 26800039 PMC4723046

[29] Tuskan GA, DiFazio S, Jansson S, Bohlmann J, Grigoriev I, Hellsten U, et al The genome of black cottonwood *Populus trichocarpa* (Torr. & Gray). Science, 2006; 313: 1596–1604. 10.1126/science.1128691 16973872

[30] Huang DI, Hefer CA, Kolosova N, Douglas C, Cronk Q. Whole plastome sequencing reveals deep plastid divergence and cytonuclear discordance between closely related balsam poplars, *Populus balsamifera* and *P*. *trichocarpa* (Salicaceae). New Phytologist, 2014; 204: 693–703. 10.1111/nph.12956 25078531

[31] Zhang QJ, Gao LZ. The complete chloroplast genome sequence of desert poplar (*Populus euphratica*). Mitochondrial DNA Part A. 2016; 27: 721–723. 10.3109/19401736.2014.913159 24810062

[32] Yang JB, Li DZ. Li HT. Highly effective sequencing whole chloroplast genomes of angiosperms by nine novel universal primer pairs. Mol. Ecol. Resour. 2014; 14: 1024–1031. 10.1111/1755-0998.12251 24620934

[33] Schattner P, Brooks AN, Lowe TM. The tRNAscan-SE, snoscan and snoGPS web servers for the detection of tRNAs and snoRNAs. Nucleic. Acids. Res. 2005; 33: 686–689. 10.1093/nar/gki366 PMC1160127 15980563

[34] Lohse M, Drechsel O, Bock R. Organellar Genome DRAW (OGDRAW): a tool for the easy generation of high-quality custom graphical maps of plastid and mitochondrial genomes. Curr. Genet. 2007; 52: 267–274. 10.1007/s00294-007-0161-y 17957369

[35] Kumar S, Nei M, Dudley J, Tamura K. MEGA: biologist centric software for evolutionary analysis of DNA and protein sequences. Brief Bioinformatics. 2008; 9: 299–306. 10.1093/bib/bbn017 18417537 PMC2562624

[36] Rosenberg MS, Subramanian S, Kumar S. Patterns of transitional mutation biases within and among mammalian genomes. Mol. Biol. Evol. 2003; 20: 988–993 10.1093/molbev/msg113 12716982

[37] Thiel T, Michalek W, Varshney R, Graner A. Exploiting EST databases for the development and characterization of gene-derived SSR markers in barley (*Hordeum vulgare* L.). Theor. Appl. Genet. 2003; 106: 411–422. 10.1007/s00122-002-1031-0 12589540

[38] Kurtz S, Choudhuri JV, Ohlebusch E, Schleiermacher C, Stoye J, Giegerich R. REPuter: the manifold applications of repeat analysis on a genomic scale. Nucleic Acids Res. 2001; 29: 4633–4642. 10.1093/nar/29.22.4633 11713313 PMC92531

[39] Katoh K, Standley DM. MAFFT multiple sequence alignment software version 7: improvements in performance and usability. Mol. Biol. Evol. 2013; 30: 772–780. 10.1093/molbev/mst010 23329690 PMC3603318

[40] Librado P, Rozas J. Dnasp v5: a software for comprehensive analysis of DNA polymorphism data. Bioinformatics. 2009; 25: 1451–1452. 10.1093/bioinformatics/btp187 19346325

[41] Chen ZY, Wang WW, Yang WL, Ma T. Characterization of the complete chloroplast genome of *Populus ilicifolia*. Conservation Genet. Resour. 2016; 8: 1–3.

[42] Fan LQ, Hu H, Zheng HL, Wang TJ, Wang YL, Ma T, et al Complete sequence and comparative analysis of the chloroplast genome of the Chinese aspen (*Populus adenopoda*, Salicaceae). Journal of Sichuan University (Natural science edition). 2018; 55(1): 165–171.

[43] Wang TJ, Fan LQ, Guo XL, Wang K. Characterization of the complete chloroplast genome of *Populus qiongdaoensis* T. Hong et P. Luo. Conservation Genet. Resour. 2016; 8: 435–437.

[44] Zheng HL, Fan LQ, Wang TJ, Zhang L, Ma T, Mao KS. The complete chloroplast genome of *Populus rotundifolia* (Salicaceae). Conservation Genet. Resour. 2016; 8: 1–3.

[45] Stamatakis A. RAxML-VI-HPC: maximum likelihood-based phylogenetic analyses with thousands of taxa and mixed models. Bioinformaties. 2006; 22: 2688–2690. 10.1093/bioinformatics/btl446 16928733

[46] Ronquist F, Huelsenbeck JP. Mrbayes 3: Bayesian phylogenetic inference under mixed models. Bioinformatics. 2003; 19: 1572–1574. 10.1093/bioinformatics/btg180 12912839

[47] Darriba D, Taboada GL, Doallo R, Posada D. jModelTest 2: more models, new heuristics and parallel computing. Nat. Methods. 2012; 9: 772. 10.1038/nmeth.2109 PMC4594756 22847109

[48] Zhang QJ, Gao LZ. The complete chloroplast genome sequence of desert poplar (*Populus euphratica*). Mitochondrial DNA. 2016; 27: 721 10.3109/19401736.2014.913159 24810062

[49] Han XM, Wang YM, Liu YJ. The complete chloroplast genome sequence of *Populus wilsonii* and its phylogenetic analysis. Mitochondrial DNA Part B. 2017; 2: 932–933. 10.1080/23802359.2017.1413291 PMC7799498 33474042

[50] Palmer JD, Osorio B, Aldrich J, Thompson WF. Chloroplast DNA evolution among legumes: Loss of a large inverted repeat occurred prior to other sequence rearrangements. Curr. Genet. 1987; 11: 275–286.

[51] Palmer JD. Plastid chromosomes: Structure and evolution In Molecular Biology of Plastids; Bogorad L. Ed. Academic Press: San Diego, CA, USA, 1991; 5–53.

[52] Wang Y, Zhan DF, Jia X, Mei WL, Dai HF, Chen XT, et al Complete chloroplast genome sequence of *Aquilaria sinensis* (Lour.) Gilg and evolution analysis within the Malvales order. Front. Plant Sci. 2016; 7: 280 10.3389/fpls.2016.00280 27014304 PMC4781844

[53] Sharp PM, Li W.H., 1986 An evolutionary perspective on synonymous codon usage in unicellular organisms. J. Mol. Evol. 24, 28–38. 3104616 10.1007/BF02099948

[54] Zhao J, Qi B, Ding L, Tang X. Based on RSCU and Qrscu research codon bias of F/10 and G/11 xylanase. J. Food Sci. Biotechnol. 2010; 29: 755–764.

[55] Zuo LH, Shang AQ, Zhang S, Yu XY, Ren YC, Yang MS, et al The first complete chloroplast genome sequences of *Ulmus* species by de novo sequencing: Genome comparative and taxonomic position analysis. 2017; PLoS ONE 12: e0171264 10.1371/journal.pone.0171264 28158318 PMC5291543

[56] Dong WP, Liu H, Xu C, Zuo YJ, Chen ZJ, Zhou SL. A chloroplast genomic strategy for designing taxon specific DNA mini-barcodes: a case study on ginsengs. BMC Genetics. 2014; 15: 138 10.1186/s12863-014-0138-z 25526752 PMC4293818

[57] Kaur S, Panesar PS, Bear MB, Kaur V. Simple sequence repeat markers in genetic divergence and marker-assisted selection of rice cultivars: A review. Crit. Rev. Food Sci. Nutr. 2015; 55: 41–49. 10.1080/10408398.2011.646363 24915404

[58] Huang J, Chen RH, Li XG. Comparative analysis of the complete chloroplast genome of four known *Ziziphus* species. Genes 2017; 8: 340. 10.3390/genes8120340 PMC5748658 29186778

[59] Kuang DY, Wu H, Wang YL, Gao LM, Zhang SZ, Lu L. Complete chloroplast genome sequence of *Magnolia kwangsisensis* (Magnoliaceae): implication for DNA barcoding and population genetics. Genome 2011; 54: 663–673. 10.1139/G11-026 21793699

[60] Qian J, Song J, Gao H, Zhu Y, Xu J, Pang X. The complete chloroplast genome sequence of the medicinal plant *Salvia miltiorrhiza*. PLoS ONE 2013; 8: e57067 10.1371/journal.pone.0057067 23460883 PMC3584094

[61] Zhang YJ, Du LW, Liu A, Chen JJ, Wu L, Hu WM, et al The complete chloroplast genome sequences of five *Epimedium* species: Lights into phylogenetic and taxonomic analysis. 2016; Front. Plant Sci. 7: 696 10.3389/fpls.2016.00696 27014326 PMC4791396

[62] CavalierSmith T. Chloroplast evolution: Secondary symbiogenesis and multiple losses. Curr. Biol. 2002; 12: 62–64. 10.1016/s0960-9822(01)00675-3 11818081

[63] Gao L, Yi X, Yang YX, Su Y, Wang T. Complete chloroplast genome sequence of a tree fern Alsophila spinulosa: insight into evolutionary changes in fern chloroplast genomes. BMC Evol. Bio. 2009; 9: 130. 19519899 10.1186/1471-2148-9-130 PMC2706227

[64] Nie XJ, Lv SZ, Zhang YX, Du XH, Wang L, Biraadar SS, et al Complete chloroplast genome sequence of a major invasive species, crofton weed (*Ageratina adenophora*). PLoS ONE 2012; 7: e36869 10.1371/journal.pone.0036869 22606302 PMC3350484

[65] Kress WJ, Wurdack KJ, Zimmer EA, Weigt LA, Janzen DH. Use of DNA barcodes to identify flowering plants. Proc. Nat. Acad. Sci. USA. 2005; 102: 8369–8374. 10.1073/pnas.0503123102 15928076 PMC1142120

[66] Makalowski W, Boguski MS. Evolutionary parameters of the transcribed mammalian genome: an analysis of 2820 orthologous rodent and human sequences. Proc. Natl. Acad.Sci. USA. 1998; 95: 9407–9412. 10.1073/pnas.95.16.9407 9689093 PMC21351

[67] Grove CE, Yu Y, Wing RA, Paterson AH, Wendel JF. A phylogenetic analysis of indel dynamics in the cotton genus. Mol. Biol. Evol. 2008; 25: 1415–1428. 10.1093/molbev/msn085 18400789

[68] Bapteste E, Philippe H. The potential value of indels as phylogenetic markers: position of trichomonads as a case study. Mol. Biol. Evol. 2001; 19: 972–977. 10.1093/oxfordjournals.molbev.a004156 12032255

[69] Simmons MP, Ochotereana H, Carr TG. Incorporation, relative homoplasy, and effect of gap characters in sequence-based phylogenetic analysis. Syst. Biol. 2001; 50: 454–462. 12116587

[70] Yang Z, Yoder AD. Estimation of the transition/transversion rate bias and species sampling. J. Mol. Evol. 1999; 48: 274–283. 10093216 10.1007/pl00006470

[71] Kim KJ, Lee HL. Wide spread occurrence of small inversions in the chloroplast genomes of land plants. Mol. Cells. 2005; 19: 104–113. 15750347

[72] Santiago AC, Beatriz OS, Juan C V. Evolution of small inversions in chloroplast genome: a case study from a recurrent inversion in angiosperms. Cladistics. 2009; 25: 93–104. 10.1111/j.1096-0031.2008.00236.x 34879620

[73] Yang YC, Zhou T, Duan D, Yang J, Feng L, Zhao GF. Comparative analysis of the complete chloroplast genomes of five *Quercus* species. Front. Plant Sci. 2016; 7: 959 10.3389/fpls.2016.00959 27446185 PMC4923075

[74] Moore MJ, Bell CD, Soltis PS, Soltis DE. Using plastid genome-scale data to resolve enigmatic relationships among basal angiosperms. Proc. Natl. Acad. Sci. USA 2007; 104: 19363–19368. 10.1073/pnas.0708072104 18048334 PMC2148295

[75] Li X, Zhang TC, Qian Q, Ren Z, Zhao J, Yonezawa T, et al Complete chloroplast genome sequence of holoparasite *Cistanche deserticola* (Orobanchaceae) reveals gene loss and horizontal gene transfer from its host *Haloxylon ammodendron* (Chenopodiaceae). PLoS ONE 2013; 8(3): e58747 10.1371/journal.pone.0058747 23554920 PMC3598846

[76] Fang Z. F., Zhao S. D., and Skvortsov A. K. (1999). Flora of China (English Version). Beijing: Science press, 162–274.

[77] Zhang D, Zhang Z, Yang K. Identification of AFLP markers associated with embryonic root development in *Populus tomentosa*. Silvae Genet. 2007; 56: 27–32.

[78] He CZ. Study on genetic diversity and origin of Populus tomentosa Carr. PhD Thesis. Beijing Forestry University. 2005.

[79] Wang DS, Wang ZS, Du SH, Zhang JG. Phylogeny of section *Leuce* (*Populus*, Salicaceae) inferred from 34 chloroplast DNA fragments. Biochem. Syst. Ecol. 2015; 63: 212–217.

[80] Wang DS, Wang ZS, Kang XY, Zhang JG. Genetic analysis of admixture and hybrid patterns of *Populus hopeiensis* and *P*. *tomentosa*. Scientific Reports. 2019, 9: 4821 10.1038/s41598-019-41320-z 30886279 PMC6423230

[81] Yin J.Y. Study on phylogeny and relationships of the genus Populus natural strand in Ergis River watershed. PhD Thesis. Chinese Academy of Forestry. 2006;

